# Microarray Analysis of Bacterial Gene Expression: Towards the Regulome

**DOI:** 10.1002/cfg.193

**Published:** 2002-08

**Authors:** Sharon L. Kendall, Farahnaz Movahedzadeh, Andreas Wietzorrek, Neil G. Stoker

**Affiliations:** Department of Pathology and Infectious Diseases Royal Veterinary College Royal College Street London NW1 0TU UK

## Abstract

Microarray technology allows co-regulated genes to be identified. In order to identify genes that are controlled by specific regulators, gene expression can be compared in mutant and wild-type bacteria. However, there are a number of pitfalls with this approach; in particular, the regulator may not be active under the conditions in which the wild-type strain is cultured. Once co-regulated genes have been identified, proteinbinding motifs can be identified. By combining these data with a map of promoters, or operons (the operome), the regulatory networks in the cell (the regulome) can start to be built up.


**Comparative and Functional Genomics**
*Comp Funct Genom* 2002; **3**: 352–354.
Published online in Wiley InterScience (www.interscience.wiley.com). **DOI:** 10.1002/cfg.193

**Conference Review**

### Microarray analysis of bacterial gene expression: towards the regulome

Sharon L. Kendall,* Farahnaz Movahedzadeh, Andreas Wietzorrek and Neil G. Stoker
*Department of Pathology and Infectious Diseases, Royal Veterinary College, Royal College Street, London NW1 0TU, UK*

**Correspondence to: Sharon L. Kendall, Department of Pathology and Infectious Diseases, Royal Veterinary College, Royal College Street, London NW1 0TU, UK. E-mail: skendall@rvc.ac.uk*

Received: 31 May 2002
Accepted: 10 June 2002

### Abstract

**Microarray technology allows co-regulated genes to be identified. In order to identify genes that are controlled by specific regulators, gene expression can be compared in mutant and wild-type bacteria. However, there are a number of pitfalls with this approach; in particular, the regulator may not be active under the conditions in which the wild-type strain is cultured. Once co-regulated genes have been identified, protein-binding motifs can be identified. By combining these data with a map of promoters, or operons (the operome), the regulatory networks in the cell (the regulome) can start to be built up. Copyright © 2002 John Wiley & Sons, Ltd.**

**Keywords:** ***Mycobacterium tuberculosis*; regulon; two-component regulatory system; promoter analysis; DNA-binding motif; mutagenesis; operome; RNA**

### Introduction

The completion of genomic sequences has revolutionized the way we look at the genetics of microorganisms. The locations of genes can be predicted with a high degree of certainty, although occasional small changes will occur, such as the identification of a new small gene, or the reassignment of the start codon for a gene. The challenge now is to determine the regulatory networks that lead to different patterns of gene expression, sometimes (inevitably) called the *regulome*.

Characterizing the regulome is difficult even in relatively well-characterized organisms; it is much more challenging in 'non-model organisms', such as our own particular interest, *Mycobacterium tuberculosis*. One place to start is with the regulatory genes, many of which can be identified as transcription factors through homology. Thus, *M. tuberculosis* is predicted to have 13 sigma factors and over 100 transcriptional regulators [1]. However, it is not usually possible to identify the sequences these regulate through homology, unless it can be shown that the binding site is conserved with one that has been characterized elsewhere. Thus, in mycobacteria, the iron regulator IdeR binds to a site similar to that recognized by *Corynebacterium diphtheriae* DtxR [8], and the *sigH* motif is related to the *sigR* motif in *Streptomyces coelicolor* [7]. It is less common for such motifs to be conserved between widely divergent species, so model organisms such as *E. coli* may be of little use. Where there is extra evidence to suggest orthology with a well-characterized system, likely regulatory sites can be propsed, e.g. the *M. tuberculosis kdpE* regulator lies next to the *kdpFABC* operon, and it is likely that it controls these genes, as in *E. coli* [10].

We have been studying two-component regulatory systems (2CRs) [4,9], which consist of a membrane spanning sensor and a cytoplasmic transcription factor. The sensors can be thought of as messengers, informing the inside of the cell of the conditions outside. When a relevant condition changes (e.g. pH or oxygen tension), the sensors respond by transferring a phosphate group to the transcription factor. This alters the DNA binding properties of the transcription factor and ultimately results in the down-regulation or up-regulation (or both) of a set of genes under its control (its regulon).

Copyright © 2002 John Wiley & Sons, Ltd.

### Experimental approaches

Experimentally, microarrays provide a way to identify the regulon. A simple approach is to inactivate a regulator, then to compare expression in mutant and wild-type. This should be more focused than exposing bacteria to general stresses, which may activate several systems. However, there are a number of caveats:

- The changes seen will be a mixture of direct and indirect effects, e.g. a regulator may activate a second regulator, leading to a cascade. Therefore it will be necessary to carry out further experiments, preferably demonstrating protein binding, to confirm direct effects.
- It is common to see a large number of slight alterations in gene expression, which are hard to account for. The same genes are frequently seen up- or down-regulated, e.g. a comment was made that expression of the *acr* gene of *M. tuberculosis* will change if you sneeze near the culture! This may reflect the effects of subtle stresses placed on the cell that are hard to determine. Of course, as the expression of these genes becomes better understood, we may be able to identify what these stresses are.
- It is important that the strains are grown under relevant conditions; if the regulator is not expressed in the wild-type strain, no difference will be seen when compared to mutant. A possible way round this is to overproduce the regulator in a mutant lacking the cognate sensor. Under these conditions the system is always switched on, even in the absence of an environmental signal, and this has been used to determine several regulons in *Bacillus subtilis* [5].
- Levels of RNA are higher at the start of operons than at the end; this may be a real effect, or it may be due to unequal degradation. Expression of genes at the end of an operon may even appear unchanged due to this phenomenon.
- It is possible for induced transcription of one gene to continue into an adjacent gene before termination occurs. If this transcription extends over the site of the target probe of the second gene, there will be apparent induction that is completely artifactual. It would help for software packages used in the analysis and visualization of microarray data to contain information on the orientation of the genes. This artifact will also be less of a problem where oligonucleotide arrays are used.
- Cross-hybridization between homologous sequences may cause incorrect signals.
- Many genes show very low levels of expression; as ratios are used in the analyses, levels of apparent up- and down-regulation are particularly volatile; even if a change is real, it might be difficult to obtain significant data with such genes, and a different technique might be needed.

An alternative approach is to apply a stress, identify co-regulated genes, and then to try to determine which are regulated by the same protein. A different problem encountered in this case is how to identify the regulatory protein.

### Motif searching

DNA binding proteins bind to promoters in a sequence-dependent way, and these short sequence motifs may be described as a profile, where different weightings are given to bases at different positions. There is tremendous scope, therefore, for integrating a bioinformatics approach with experimental work. Experimental work can be used to identify co-regulated sequences, with common motifs being identified through statistical analysis. The resulting profile can then be used to search the genome for other potential sites, and the DNA-binding properties tested experimentally. The availability of closely related genomes is particularly powerful in this respect, as protein-binding sites evolve less quickly than other non-coding sequences [2,3,6].

### A promoter map — the operome

One tool which would be highly useful for these analyses would be a promoter map. In bacteria, this is particularly important because of the presence of operons, with promoters signalling the start of a polycistronic mRNA. We use the term 'operome' for this. A promoter (or operon) map would allow:

- Validation of microarray patterns against this map.
- Identification of relevant promoter regions for motif analysis.

Copyright © 2002 John Wiley & Sons, Ltd. 

It would also provide the basis of a regulon map, which could then start to be tested and built up as more data appears.

### Acknowledgements

We acknowledge BμG@S (the Bacterial Microarray Group at St George's) and The Wellcome Trust for funding this work. This work was financed by The Wellcome Trust (Grant No. 062508), the EU programme (Grant No. QLK2-CT-1999-01093) and GlaxoSmithKline (Grant No. G1913).

### References

1. Cole ST, Brosch R, Parkhill J, *et al.* 1998. Deciphering the biology of *Mycobacterium tuberculosis* from the complete genome sequence. *Nature* **393**: 537–544.
2. Gelfand MS, Koonin EV, Mironov AA. 2000. Prediction of transcription regulatory sites in Archaea by a comparative genomic approach. *Nucleic Acids Res* **28**: 695–705.
3. Gelfand MS, Novichkov PS, Novichkova ES, Mironov AA. 2000. Comparative analysis of regulatory patterns in bacterial genomes. *Brief Bioinform* **1**: 357–371.
4. Hoch JA. 2000. Two-component and phosphorelay signal transduction. *Curr Opin Microbiol* **3**: 165–170.
5. Kobayashi K, Ogura M, Yamaguchi H, *et al.* 2001. Comprehensive DNA microarray analysis of *Bacillus subtilis* two-component regulatory systems. *J Bacteriol* **183**: 7365–7370.
6. Manson McGuireA, Church GM. 2000. Predicting regulons and their cis-regulatory motifs by comparative genomics. *Nucleic Acids Res* **28**: 4523–4530.
7. Paget MS, Molle V, Cohen G, Aharonowitz Y, Buttner MJ. 2001. Defining the disulphide stress response in *Streptomyces coelicolor* A3(2): identification of the sigmaR regulon. *Mol Microbiol* **42**: 1007–1020.
8. Schmitt MP, Predich M, Doukhan L, Smith I, Holmes RK. 1995. Characterization of an iron-dependent regulatory protein (IdeR) of *Mycobacterium tuberculosis* as a functional homolog of the diphtheria toxin repressor (DtxR) from *Corynebacterium diphtheriae*. *Infect Immun* **63**: 4284–4289.
9. Stock AM, Robinson VL, Goudreau PN. 2000. Two-component signal transduction. *Ann Rev Biochem* **69**: 183–215.
10. Walderhaug MO, Polarek JW, Voelkner P, *et al.* 1992. KdpD and KdpE, proteins that control expression of the KdpABC operon, are members of the 2-component sensor-effector class of regulators. *J Bacteriol* **174**: 2152–2159.

Copyright © 2002 John Wiley & Sons, Ltd. 
